# Could Environment Affect the Mutation of H1N1 Influenza Virus?

**DOI:** 10.3390/ijerph17093092

**Published:** 2020-04-29

**Authors:** Dong Jiang, Qian Wang, Zhihua Bai, Heyuan Qi, Juncai Ma, Wenjun Liu, Fangyu Ding, Jing Li

**Affiliations:** 1 State Key Laboratory of Resources and Environmental Information System, Institute of Geographical Sciences and Natural Resources Research, Chinese Academy of Sciences, Beijing 100101, China; jiangd@igsnrr.ac.cn (D.J.); wangq.17s@igsnrr.ac.cn (Q.W.); 2 Savaid Medical School, University of Chinese Academy of Sciences, Beijing 100049, China; baizhihua19@mails.ucas.ac.cn (Z.B.); liuwj@im.ac.cn (W.L.); 3 CAS Key Laboratory of Pathogenic Microbiology and Immunology, Institute of Microbiology, Chinese Academy of Sciences, Beijing 100101, China; qihy@im.ac.cn (H.Q.); ma@im.ac.cn (J.M.); 4 State Key Laboratory for Conservation and Utilization of Subtropical Agro-Bioresourses & Laboratory of Animal Infectious Diseases, College of Animal Sciences and Veterinary Medicine, Guangxi University, Nanning 530004, Guangxi, China

**Keywords:** H1N1 influenza virus, mutation, environment factors

## Abstract

H1N1 subtype influenza A viruses are the most common type of influenza A virus to infect humans. The two major outbreaks of the virus in 1918 and 2009 had a great impact both on human health and social development. Though data on their complete genome sequences have recently been obtained, the evolution and mutation of A/H1N1 viruses remain unknown to this day. Among many drivers, the impact of environmental factors on mutation is a novel hypothesis worth studying. Here, a geographically disaggregated method was used to explore the relationship between environmental factors and mutation of A/H1N1 viruses from 2000–2019. All of the 11,721 geo-located cases were examined and the data was analysed of six environmental elements according to the time and location (latitude and longitude) of those cases. The main mutation value was obtained by comparing the sequence of the influenza virus strain with the earliest reported sequence. It was found that environmental factors systematically affect the mutation of A/H1N1 viruses. Minimum temperature displayed a nonlinear, rising association with mutation, with a maximum ~15 °C. The effects of precipitation and social development index (nighttime light) were more complex, while population density was linearly and positively correlated with mutation of A/H1N1 viruses. Our results provide novel insight into understanding the complex relationships between mutation of A/H1N1 viruses and environmental factors.

## 1. Introduction

Influenza viruses possess an RNA genome and belong to the *Orthomyxoviridae* family [1]. Segmenting genes attributed to influenza viruses, when two or more different influenza virus strains infect the same host or cell an antigen shift may occur due to gene recombination. Theoretically, recombination of two different strains of influenza virus will produce a variety of influenza viruses. Therefore, gene recombination is an important reason for the diversity of the virus. Replacement and accumulation of surface glycoprotein genes leads to antigenic drift and, thus, the outbreak and epidemic of influenza each year. This diversity enables the rapid evolution of influenza viruses, their adaptation to new host environments, their evasion from the host’s immune response, and the accelerated generation of drug-resistant strains [2]. Of course, the evolution of influenza viruses has required constant changes in the composition of vaccines for seasonal influenza and for the continued development and preparation of candidate vaccine strains for pandemic prevention and control [3,4]. H1N1 subtype influenza A viruses have a major impact on the epidemiology of humans by causing seasonal epidemics of different degrees of severity and two pandemics in 1918 and 2009 [5]. Though complete genome sequences have recently been reported, the evolution, epidemiology, transmission dynamics, and other aspects of A/H1N1 viruses remain unknown to this day.

Over the past few decades, global climate change and the integration of economic development in urban and rural areas have greatly impacted Earth’s ecology. Climate change has significant impacts on both human migration and population health, especially infectious disease. It has not only a direct impact on the ecology of infectious diseases, but also a remote impact on the ability of society to control and prevent diseases [6,7]. Christine et al. demonstrate the strong regulating effect of temperature on microbial diversity through 16S rRNA gene sequencing [8]. Although the change in diversity at the gene level is of greater concern, the effect of these factors on the gene mutation of the influenza viruses is still not clear. According to the research of Aitor Nogales et al., the H3N2 virus encoding the NS1-V194I protein displays a temperature-sensitive phenotype, providing an assumption of the effect of environmental factors such as temperature on the genetic mutation of influenza viruses [9]. Furthermore, as urbanization and population density have increased, seasonal infectious diseases are more difficult to prevent and treat [10]. In terms of social factors, Chris et al. used the theory of games to study the impact of social collaboration and human vaccination on epidemics. Human social behavior is closely related to the emergence of influenza virus resistance and the occurrence of infectious diseases [11,12]. Jan proposed that the fitness penalty of pathogens could be used as a predictor of the durability of disease resistance genes [13]. However, there are still many unknowns surrounding the safety of antiviral drugs and effectiveness of that strategy. It is important to collect data on climate, urbanization, socio-economic conditions, and virus incidence rate, which is helpful in understanding the interaction between climate change, urbanization and public health, as well as in adequately planning for the future. 

In the present study, the location information (i.e., latitude and longitudes) of 11,721 reported cases of H1N1 were collected and we explored if H1N1 genomic diversity was subject to the direct effects of temperature, precipitation, and other natural factors or the indirect effects of population density, urban development, and other social factors.

## 2. Methods

### 2.1. Outcome Data

The H1N1 influenza data reported on the Chinese mainland from the World Health Organization (WHO)’s Global Influenza Program (GIP, http://who.int/influenza/) were collected. The H1N1 dataset included 12,401 records from the first case reported in 1931 to the last case reported in 2019. The datasets included the basic information (reported province, reported date, latitude, and longitude) for each case (Supplementary Table S1). 

### 2.2. HA Mutation

The sequences of influenza virus strains were compared with those of the earliest reported strain and the sequences of other strains to obtain the location and number of different sequences. The number of differentially expressed genes was taken as the numerator, and the total number of strains with the earliest reported was taken as the denominator to calculate the difference rate, i.e., the mutation rate of the HA gene.

### 2.3. Environmental Variables

Biological experiments and epidemiological evidence indicate that variations in environment have important effects on the occurrence and transmission of epidemic influenza. There are many reports demonstrating that the spread of influenza virus is closely related to the two climatic factors of temperature and precipitation: low-temperature dry environments are conducive to the spread of the virus, and high-temperature environments can largely prevent the virus from spreading in aerosols. However, there are few studies on how these two factors affect the mutation of the virus. The temperature and precipitation data used in this experiment were downloaded from the WorldClim database (version 2.0, http://www.wordclim.org) with a spatial resolution of 1 × 1 km^2^ (Table 1). The temperature and precipitation values were obtained corresponding to each case point by ArcGIS processing.

### 2.4. Socio-Economic Variables

Population density, urban accessibility, and nighttime light were chosen as social factors simulating the impact of mutation, and urbanicity was added as a fixed factor of the model. Population, economy, and transportation are three important factors that reflect social conditions, and urbanicity is a more direct factor for distinguishing the level of social development. To a certain extent, urban accessibility reflects the spatial distribution and traffic conditions of an area. Many studies show that, as an effective representation of human activities, nighttime light data has a significant correlation with GDP and can be used to invert regional GDP development. As the host of the virus, the higher the population density is, the faster the virus spreads, so this is reason to believe that population density has a certain relationship with virus transmission and mutation.

The urban accessibility data was obtained with a spatial resolution of 1 km × 1 km from the European Commission Joint Research Center Global Environment Monitoring Unit, (https://ec.europa.eu/info/departments/joint-research-centre_en). The population density data was downloaded from Socioeconomic Data and Applications (Table 1), https://sedac.ciesin.columbia.edu/. The DMSP-OLS Nighttime light data was used from the Earth Observation Group, NOAA, and the fixed factor urbanicity data from the National Aeronautics and Space Administration (NASA). The spatial resolution of all these social variables was 1 km × 1 km, and these data were included in our case dataset by using both the geographical locations assigned and the temporal period at a decadal resolution via ArcGIS 10.2 made by Environmental Systems Research Institute of America.

### 2.5. Year Variables

The years variable was obtained by using the year in which a case occurred minus the year 1931 in which H1N1 was first reported, and this variable ranged from 69 to 88.

### 2.6. Statistical Analyses

All data coordinates were unified as WGS-84. GLM with a Gaussian distribution was used to study the association between environment and social conditions and H1N1 mutation (Table 2, column a). It was found that most of the elements were not significantly related to mutation, so a more flexible model (with nonlinear parameters) was required.

**Figure 1:**
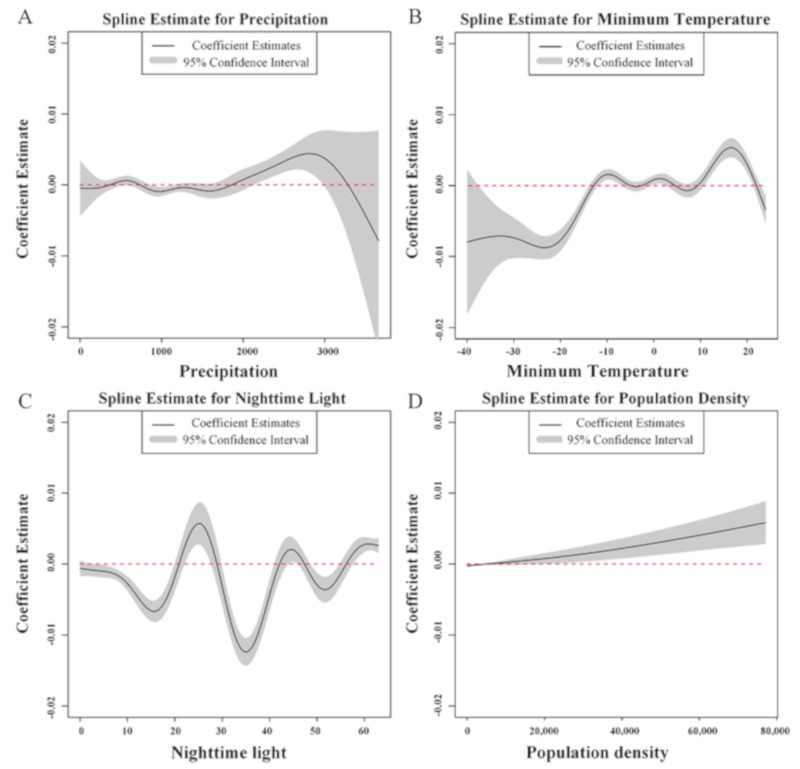
Plots showing the coefficient estimate and 95% confidence interval over the range of precipitation (**A**), minimum temperature (**B**), nighttime light (**C**), and population density (**D**) for the model in Table 2, column b. Non-overlap between the confidence interval and dashed zero line indicates a statistically significant effect.

To address this nonlinearity, a GAM using the R package mgcv and a thin-plate spline for several factors were estimated (Table 2, column b). This specification allowed factor coefficients to vary over the values within their distributions, and it enabled us to explore the nuances of the relationship between our measurements and HA mutation. Since these spline variables do not have a single coefficient estimate, the coefficients for some factors were presented graphically (Figure 1).

For both the GLM and GAM versions of the models, we controlled for residual unmeasured regional differences by using the urban-rural partition variable. This urban-rural variable was included as a fixed, instead of random, effect attributed to the fact that several of the predictor variables are reported differently in rural and urban areas. Therefore, they are correlated at that spatial scale. The total number of virus strains was also included as another fixed factor to avoid different response power to environmental factors caused by different virus strains. The GAM version of the model has the following functional form:

(1) $$Y_{\mathrm{it}}=a+\sum_{k}^{k=7}f_{it}{\left(X_{it}\right)}_{k}+Urban_{it}+M_{it}+\varepsilon_{it}$$

where *i* = case, *t* = time, *Y* is HA mutation, parameter *a* is the overall intercept, *X* is the independent variable, *f* ( ) is the thin-plate spline function, Urban is the fixed effect term urban-rural variable, *M* is the fixed factor total number of virus strains, and ε is the error term.

## 3. Results

### H1N1 HA Mutation

**Figure 2:**
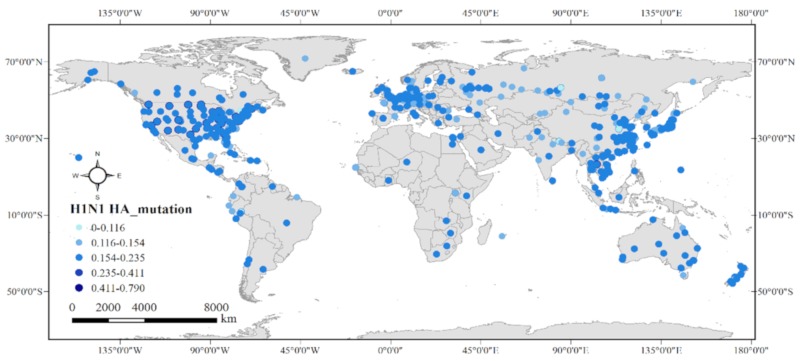
Spatial distribution of H1N1 cases from 2000–2019 distinguished by color according to their HA mutation.

Figure 2 shows the spatial distribution of H1N1 cases from 2000–2019, totaling 11,721 reported infections. The cases spatially clustered in three major areas of the world during this timeframe: the southern portion of North America; central Europe; and Southeast Asia. Meanwhile, there was sporadic distribution in Oceania, South America, and Africa. In North America, cases were mainly distributed in the USA, the country with the most cases in the world, accounting for 57.7% of the total. The United Kingdom, Finland, and New Zealand were the main affected areas in Europe. In Asia, cases were concentrated in the southeast, such as Singapore, Thailand, and China’s southeastern cities.

In terms of HA mutation value, in all 11,721 cases the boundary of the variability was very obvious: 46 were > 0.75, two were between 0.55 and 0.60, and all of the remaining were < 0.22. At the country level, cases with high mutation value occurred only in two countries, the United States (44/46) and the United Kingdom (2/46). Cases with low values, < 0.01, were mostly distributed in Europe and Russia, and 26 of the 37 were located in the Netherlands.

For H1N1’s HA mutation from 2000–2019, a simple generalized linear model (GLM) shows that, of all the seven independent variables, most variables displayed a strong significance with HA mutation, including maximum temperature, minimum temperature, nighttime light, population density, and years fixed factor. Precipitation and urban accessibility were not statistically significant in the basic GLM model (Table 2, column a). To capture any possible coefficient variations over the variable range, estimates in a generalized additive model (GAM) were allowed to vary (Table 2, column b). The results of the GAM model show that the relationship of the five above variables and HA mutation was still significant, and the connection between nighttime light and mutation became very significant. At the same time, two variables (precipitation and urban accessibility) displayed significant association with mutation in the GAM but not GLM model.

Here, AIC (Akaike Information Criterion) and explained deviance were used to consider the simulation effect of the model. The AIC of GAM is lower than that of GLM, −68,857.5 vs. −67,655.2. The deviance explained of GAM is 90.5%, while that of GLM is 89.4%. These results indicate that the simulation capability of the GAM model is better for this experimental process.

Figure 1 shows the impact of four variables on mutation, with precipitation (A) and minimum temperature (B) being selected as representatives of environmental factors, and nighttime light (C) and population density (D) as representative social factors. In terms of the relationship between precipitation and mutation (Figure 1A), the mutation rate does not change much with an increase of precipitation within about 2000 mm. Above 2000 mm, the rate of mutation first increases and then decreases with the increase in precipitation, but overall the effect is not significant. Minimum temperature displayed a nonlinear, rising association with HA mutation, with a maximum around 15 °C (Figure 1B). The curve as a whole is first stable, then increases, and after maintaining a stable period suddenly rises and then falls. The broad effect of nighttime light on HA mutation forms an undulating wave with three troughs and two peaks (Figure 1C) and reached a minimum around 35. The impacts of the rising section are 15–25, 35–45, and > 50, with a maximum around 25. The association of population density with mutation is simpler than the above three factors, displaying a linear, positive association with mutation (Figure 1D).

## 4. Discussion

The dependence of influenza virus transmission on environmental factors, including temperature, humidity, and atmospheric pressure, has been documented by many previous studies [14,15,16,17]. Therefore, the question arises as to whether these climatic factors are related to influenza virus mutations. In this study, the correlation between climatic factors and HA protein mutation of H1N1 was examined. As for the relationship between precipitation and mutation, the result was shown that the effect on mutations only occurred when precipitation was > 2000 mm, though the relationship was not linear. However, the average annual precipitation of many countries with high rates of H1N1 subtype influenza A virus mutation, especially the United States and United Kingdom, did not exceed 2000 mm [18,19]. Thus, precipitation is not associated with HA protein mutations in a practical sense.

Another climatic factor was the minimum temperature, which is slowly rising due to the influence of human activities. In recent years, multiple studies have proposed that global warming will likely influence the life of all living species, including the evolution of influenza A virus [20,21]. Furthermore, Yan et al. found that global warming affects many different levels of biological evolution; even intracellular proteins are subject to global warming [22]. It is understandable that all biological functions are interconnected from the macro to micro level. In contrast, from the perspective of cross-species transmission of avian influenza, our previous studies on the correlation between climate factors and avian influenza infection found that there were significant relationships between climate factors and H5/H7 influenza infection, especially temperature variables [23]. Herein, the minimum temperature showed a nonlinear, rising association with HA mutation, with a maximum around 15 °C. Interestingly, Diana et al. found that the maximum values of the weekly influenza proxy coincided with the minimum temperature (10–15 °C) in the 2010–2015 influenza seasons in Spain [24]. This suggests that the highest mutation rate of HA protein at 15 °C could be related to the high incidence and transmission rate of influenza virus at ~15 °C.

Aside from meteorological factors, many other factors also affect influenza mutation rates. In terms of socioeconomic factors, nighttime light and population density were selected as the test criteria. As is known, there is less research providing information on the correlation between flu mutation rates and nighttime light. Nighttime light data can reflect comprehensive information, and depends not only on population density but also *per capita* energy consumption and, hence, economic activity [25]. Surprisingly, nighttime light and influenza mutations did not display a positive correlation as predicted but, rather, an unstable fluctuation. Considering the influence of a variety of social factors, the one potential explanation for this result is that the effect of nighttime light on HA mutation is not direct but indirect or combined with other auxiliary factors. However, these trends cannot be ignored. A better understanding of the complex effects of nighttime light will enable better prediction and manipulation of the course of influenza evolution in social contexts, so more detailed classification and analysis of various social factors are needed.

The association of population density with mutation is simpler than the above three factors, displaying a linear, positive association with mutation. This is to be expected as an excessive population can contribute to the spread of the epidemic. Notably, the continuous infection and propagation of the virus could yield genetic mutation and also affect pathogenicity and virulence [26,27]. These results indicate that interventions with a focus on municipalities with greater flows and densities of people, especially those with a higher Human Development Index and the presence of municipal air and road transport, could play an important role in mitigating the impact of future influenza pandemics on public health.

It is well known that influenza viruses display a remarkable genetic flexibility based on their high mutation rate under different selective pressures [28,29]. Our study provides an environmental perspective for understanding mutation in the evolution of the H1N1 subtype of influenza viruses. Due to its crucial role in receptor recognition and attachment, the HA protein is considered to be a principal determinant of influenza virus invasion [30,31]. Consequently, the association between HA mutations and environmental factors has been sought. In this study, a complex non-linear relationship had been found between minimum temperature, nighttime light, and HA protein mutations. Simultaneously, population density was positively correlated with HA protein mutations. These results suggest the possibility of using temperature and population density to approximate the effects that environmental factors have on H1N1 HA mutation. However, we have not analyzed and predicted the influence of these factors on the direction of HA protein mutation. Notably, much statistical data demonstrates that correlation does not mean a cause-consequence relationship. Therefore, even if correlations between two trends have not been found, whether there is any direct or indirect causation remains to be determined. Thus, wider and deeper research needs to be done. In addition, our findings showed that mutation rates of H1N1 subtype viruses were higher in the United States and the United Kingdom than in other countries, suggesting that there may be a greater risk for the emergence of novel pathogenic H1N1 strains in the US and UK. The climate and social economic risk delineated in this study should be considered as important monitoring references for the guidance of H1N1 epidemics caused by mutations.

However, it should be noted that there are several limitations in this study. For instance, the environmental variables are annual average data, which does not exactly match the virus dataset on the time scale. In the future research, we will collect macro and micro data on the same time scale as far as possible. In addition, the environmental covariates adopted in this study may not be comprehensive enough due to the availability of data. In the future research, several variables (i.e., the distance to migratory bird migration routes and the distance to water body) will be added to the statistical analysis.

## 5. Conclusions

Our findings show that environmental factors systematically affect the mutation of A/H1N1 viruses. Minimum temperature displayed a nonlinear, rising association with mutation, with a maximum ~15 °C. The effects of precipitation and social development index (nighttime light) were more complex, while population density was linearly and positively correlated with mutation of A/H1N1 viruses. This study provides a novel insight into understanding the complex relationships between mutation of A/H1N1 viruses and environmental factors, which enhances our capacity to target the potential risk areas, to develop disease control strategies and to allocate medical supplies.

## Figures and Tables

**Table 1:** Related covariates included in the analysis.

Materials	Data Source
Urban accessibility	European Commission Joint Research Center Global Environment Monitoring Unit
Population density	Socioeconomic Data and Applications Center, NASA
Urbanicity	
Nighttime light	The Earth Observation Group, NOAA
Annual cumulative precipitation	WorldClim database, version 2.0
Maximum annual temperature	
Minimum annual temperature	

**Table 2:** Regression models for HA mutation, 2000–2019.

	(a) GLM			(b) GAM Splines	
	Estimate	z-Value		Estimate	*p*-Value
(Intercept)	0.055	0.000 ***	(Intercept)	0.153	0.000
Precipitation	0.000	0.255	S (Precipitation)	7.483	0.000 ***
Maximum temperature	−0.000	0.000 ***	S (Maximum temperature)	6.160	0.000 **
Minimum temperature	0.000	0.000 ***	S (Minimum temperature)	8.886	0.000 ***
Nighttime light	−0.000	0.009 **	S (Nighttime light)	8.958	0.000 ***
Population density	0.000	0.000 ***	S (Population density)	1.440	0.005 **
Urban accessibility	−0.000	0.611	S (Urban accessibility)	8.691	0.005 *
Years	0.002	0.000 ***	S (Years)	8.973	0.000 ***
Log-likelihood	33,843.6	-	Log-likelihood	34,488.3	-
Deviance explained	89.4%	-	Deviance explained	90.5%	-
AIC	−67,655.2	-	AIC	−68,857.5	-

The number of observations for all models is 11,721. All models are estimated with urban area and HA fixed effects (not shown). GLM: Generalized Linear Model; GAM: generalized additive model; AIC: Akaike Information Criterion. Statistically significant differences are indicated (* *p* < 0.05; ** *p* < 0.01; *** *p* < 0.001).

## Supplementary Materials

The following are available online at https://www.mdpi.com/1660-4601/17/9/3092/s1, Table S1: Basic information (reported province, reported date, latitude, and longitude) for 12401 H1N1 influenza case from 1931 to 2019.
